# Supplementary material for: LUZP1, a novel regulator of primary cilia and the actin cytoskeleton, is a contributing factor in Townes-Brocks Syndrome
Source: eLife. 2020 Jun 18;9:e55957. doi: 10.7554/eLife.55957 (PMC7363444; doi:10.7554/eLife.55957)
Supplement: Supplementary file 1. [file elife-55957-supp1.docx]

| **Key Resources Table** | | | | |
| --- | --- | --- | --- | --- |
| **Reagent type (species) or resource** | **Designation** | **Source or reference** | **Identifiers** | **Additional information** |
| cell line (*Homo-sapiens*) | U2OS | ATCC | HTB-96; RRID:CVCL_0042 |  |
| cell line (*Homo-sapiens*) | HEK 293FT | Invitrogen | R70007; RRID:CVCL_6911 |  |
| cell line (*Homo-sapiens*) | hTERT-RPE1 | ATCC | CRL-4000; RRID:CVCL_4388 |  |
| cell line (*Mus musculus*) | Shh-LIGHT2 | ([Taipale et al., 2000](#_ENREF_76)) | RRID:CVCL_2721 | NIH 3T3 cell line stably incorporating the Gli-luc reporter |
| cell line (*Mus musculus*) | Luzp1^-/-^ | This work |  | The mouse *Luzp1* locus was targeted in Shh-LIGHT2 cells |
| cell line (*Mus musculus*) | +LUZP1 | This work |  | The mouse *Luzp1* locus was targeted in Shh-LIGHT2 cells and the they were transfected with EFS-LUZP1-YFP-P2A-blastR |
| biological sample (*Homo-sapiens*) | Dermal fibroblast | PMID: 29395072 | ESCTRL#2 | Freshly isolated adult female dermal fibroblasts from a healthy donor |
| biological sample (*Homo-sapiens*) | Dermal fibroblast | PMID: 29395072 | UKTBS#3 or TBS^275^ | Freshly isolated adult male dermal fibroblasts from a TBS individual |
| antibody | anti-LUZP1 (Rabbit polyclonal) | Proteintech | Cat#: 17483-1-AP; RRID:AB_2139498 | WB (1:1000) |
| antibody | anti-LUZP1 (Rabbit polyclonal) | Sigma | Cat#: HPA028506; RRID:AB_10600083 | WB (1:1000); IF (1:100); IP: 1 µg |
| antibody | anti-CCP110 (Rabbit polyclonal) | Proteintech | Cat#: 12780-1-AP; RRID:AB_10638480 | WB (1:1000); IF (1:100) |
| antibody | anti-CEP97 (Rabbit polyclonal) | Proteintech | Cat#: 22050-1-AP; RRID:AB_11182378 | WB (1:1000)  IP: 1 µg |
| antibody | anti-GFP (Mouse monoclonal) | Roche | Cat#: 11814460001; RRID:AB_390913 | WB (1:1000) |
| antibody | anti-GAPDH (Mouse monoclonal) | Proteintech | Cat#: 60004-1-Ig; RRID:AB_2107436 | WB (1:1000) |
| antibody | anti-FLNA (Mouse monoclonal) | Merck | Cat#: MABN1834 | WB (1:1000) |
| antibody | anti-BirA (Mouse polyclonal) | Sino Biological | Cat#: 11582-RP01; RRID:AB_2857347 | WB (1:1000) |
| antibody | HRP-conjugated anti-biotin  (Goat polyclonal) | Cell Signaling Technology | Cat#: 7075; RRID:AB_10696897 | WB (1:2000) |
| antibody | Anti-Myc (Mouse monoclonal) | Cell Signaling Technology | Cat#: 2276; RRID:AB_2857348 | WB (1:2000) |
| antibody | Anti-Actin (Mouse monoclonal) | Sigma | Cat#: A2228; RRID:AB_476697 | WB (1:1000) |
| antibody | Anti-GLI3 (Goat polyclonal) | R&D | Cat#: AF3690; RRID:AB_2232499 | WB (1:1000) |
| antibody | Anti-SALL1 (Mouse monoclonal) | R&D | Cat#: PP-K9814-00; RRID:AB_2183228 | WB (1:1000) |
| antibody | Anti-acetylated alpha-tubulin (Mouse monoclonal) | Santa Cruz Biotechnologies | Cat#: sc-23950; RRID:AB_628409 | IF (1:160) |
| antibody | Anti-gamma tubulin (Mouse monoclonal) | Proteintech | Cat#: 66320-1-Ig: RRID:AB_2857350 | IF (1:160) |
| antibody | Anti-Centrin-2 (Rat monoclonal) | Biolegend | Cat#: 698601; RRID:AB_2715793 | IF (1:160) |
| antibody | Anti-PCM1 (Rabbit polyclonal) | Cell signaling Technology | Cat#: 5213; RRID:AB_2857351 | IF (1:100) |
| antibody | Anti-ODF2 (Rabbit polyclonal) | Atlas Antibodies | Cat#: HPA048841; RRID:AB_2680536 | IF (1:100) |
| antibody | Anti-CEP164 (Mouse monoclonal) | Cell signaling Technology | Cat#: GTX70096; RRID:AB_2857352 | IF (1:100) |
| antibody | Anti-beta-tubulin (Mouse monoclonal) | DSHB | Cat#: AB_2315513; RRID:AB_528499 | IF (1:100) |
| antibody | Anti-Pericentrin (Mouse monoclonal) | Abcam | Cat#: ab28144; RRID:AB_2160664 | IF (1:100) |
| antibody | anti-goat HRP-conjugated | Jackson ImmunoResearch | Cat#: AB_2340390; RRID:AB_2340390 | WB (1:2000) |
| antibody | anti-mouse HRP-conjugated | Jackson ImmunoResearch | Cat#: AB_2340770; RRID:AB_2340770 | WB (1:2000) |
| antibody | anti-rabbit HRP-conjugated | Jackson ImmunoResearch | Cat#: AB_2340585; RRID:AB_2340585 | WB (1:2000) |
| antibody | Secondary: Donkey anti-rabbit polyclonal; Alexa Fluor 488-conjugated | Jackson ImmunoResearch | Cat#: AB_2313584; RRID:AB_2313584 | IF (1:200) |
| antibody | Secondary: Donkey anti-rabbit polyclonal; Alexa Fluor 594 conjugated | Jackson ImmunoResearch | Cat#: AB_2340621; RRID:AB_2340621 | IF (1:200) |
| antibody | Secondary: Donkey anti-mouse polyclonal; Alexa Fluor 594 conjugated | Jackson ImmunoResearch | Cat#: AB_2340854; RRID:AB_2340854 | IF (1:200) |
| antibody | Secondary: Donkey anti-rat polyclonal; Alexa Fluor 633 conjugated | Jackson ImmunoResearch | Cat#: AB_2340688; RRID:AB_2340688 | IF (1:200) |
| recombinant DNA reagent | *Lenti-Cas9-blast* | Addgene | Cat#: 52962; RRID:Addgene_52962 | Kind gift of F. Zhang, MIT |
| recombinant DNA reagent | *EFS-LUZP1-YFP-P2A-blastR* | This work |  | Transfected in Shh-LIGHT2 cells |
| recombinant DNA reagent | *SALL1^275^-YFP, SALL1^FL^-YFP and SALL1^FL^-2xHA* | ([Bozal-Basterra et al., 2018](#_ENREF_8)) |  | Transfected in HEK 293FT cells |
| recombinant DNA reagent | *Myc-BirA*-SALL1^275^ and Myc-BirA*-SALL1FL* | ([Bozal-Basterra et al., 2018](#_ENREF_8)) |  | Transfected in HEK 293FT cells |
| recombinant DNA reagent | *LUZP1-YFP and TbID-LUZP1* | This work |  | Vectors were generated by replacing Cas9 in Lenti-Cas9-blast (**RRID:Addgene_52962)** |
| recombinant DNA reagent | *CAG-BioUBC(x4)_BirA_V5_puro and CAG-BirA-puro* | ([Pirone et al., 2017](#_ENREF_64)) | BioUb and BirA |  |
| recombinant DNA reagent | EFS-LUZP1-YFP-P2A-blastR, EFS-YFP-P2A-blastR, LL-GFS-SALL1c.826C>T-IRES-puroR, LL-GFS-stop-IRES-puroR, EFS-TbID-LUZP1-P2A-blastR and EFS-TbID-P2A-blastR | This work |  | Lentiviral constructs transfected in Shh-LIGHT2 cells, RPE1 cells, or TBS^275^ and control human fibroblasts |
| sequence-based reagent | *LUZP1* | This work | PCR primers | LUZP1 -F:GGAATCGGGTAGGAGACACCA; LUZP1-R: TTCCCAGGCAGTTCAGACGGA |
| sequenced-based reagent | *GAPDH* | This work | PCR primers | GAPDH-F: AGCCACATCGCTCAGACAC; GAPDH-R:GCCCAATACGACCAAATCC |
| sequenced-based reagent | *Gli1* | This work | PCR primers | Gli1-F: AGCCTTCAGCAATGCCAGTGAC; Gli1-R: GTCAGGACCATGCACTGTCTTG |
| sequenced-based reagent | *Ptch1* | This work | PCR primers | Ptch1-F: AAGCCGACTACATGCCAGAG; Ptch1-R:TGATGCCATCTGCGTCTACCAG |
| sequenced-based reagent | *Rplp0* | This work | PCR primers | Rplp0-F: ACTGGTCTAGGACCCGAGAAG; Rplp0-R: CTCCCACCTTGTCTCCAGTC-3 |
| sequenced-based reagent | MmLuzp1 | This work | PCR primers | MmLuzp1_geno_for: GTTGCCAAAGAAGGTTGTGGATGCC; MmLuzp1_geno_rev:CGTAAGGTTTTCTTCCTCTTCAAGTTTCTC |
| sequenced-based reagent | CRISPR-Cas9 knockout plasmids | This work | CRISPR-Cas9 knockout plasmids | sg2: 5’-CTTAAATCGCAGGTGGCGGT_TGG-3’; sg3: 5’-CTTCAATCTTCAGTACCCGC_TGG-3’; cloned into px459 2.0; RRID:Addgene_62988 |
| commercial assay or kit | EZNA Total RNA Kit | Omega Bio-Tek | Cat. #: SKU: R6834-01 |  |
| commercial assay or kit | Effectene Transfection Reagent | Qiagen | Cat. #: 301425 |  |
| commercial assay or kit | Lipofectamine 3000 | Thermo Fisher | Cat. #: L3000001 |  |
| commercial assay or kit | Dual-Luciferase Reporter Assay System | Promega | Cat. #: E1910 |  |
| chemical compound, drug | Mimosine | Sigma Aldrich | Cat. #: M87614 | Final concentration (FC)=400µM |
| chemical compound, drug | Thymidine | Sigma Aldrich | Cat. #: T1895 | FC=2.5mM |
| chemical compound, drug | RO-3306 | Sigma Aldrich | Cat. #: SML0569 | FC=10µM |
| chemical compound, drug | MG132 | Calbiochem | Cat. #: 474790 | FC=5µM |
| chemical compound, drug | CytoD | Sigma Aldrich | Cat. #: C8273 | FC=50nM |
| chemical compound, drug | Purmorphamine | Calbiochem | Cat. #: CAS 483367-10-8 - | FC=5µM |
| chemical compound, drug | Protease inhibitor cocktail | Roche | Cat. #: 11836170001 | FC=1X |
| software, algorithm | Adobe Photoshop | Adobe Photoshop | RRID:SCR_014199 | Version: 21.0.0 |
| software, algorithm | ImageJ software | ImageJ (<http://imagej.nih.gov/ij/>) | RRID:SCR_003070 |  |
| software, algorithm | GraphPad Prism software | GraphPad Prism (https://graphpad.com) | RRID:SCR_015807 | Version 6.0 |
| software, algorithm | PEAKS software | Bioinformatics solutions (https://www.bioinfor.com/) |  |  |
| software, algorithm | Perseus platform | ([Tyanova et al., 2016](#_ENREF_79)) |  |  |
| software, algorithm | g:Profiler web server | ([Reimand et al., 2016](#_ENREF_67)) |  |  |
| software, algorithm | String app – Cytoscape | http://apps.cytoscape.org/apps/stringapp |  | Version 1.4.2 |
| other | DAPI stain | Sigma Aldrich | Cat. #: D9542 | Cf=300 ng/ml |
| other | Alexa594- conjugated Phalloidin | Invitrogen | Cat. #: A12381 | IF (1:500) |
| other | Alexa-594-conjugated Streptavidin | Jackson Immunoresearch | Cat. #: AB_2337247 | IF (1:100) |
| other | GFP booster | Chromotek | Cat. #: ABIN509419 | IF (1:500) |
| other | GFP-Trap resin | Chromotek | Cat. #: gta-10 |  |
| other | NeutrAvidin-agarose beads | Thermo Scientific | Cat. #: 29200 |  |
| other | Protein G Sepharose 4 Fast Flow beads | GE Healthcare | Cat. #: GE17-0618-01 |  |
| other | Clarity ECL | BioRad | Cat. #: 1705061 |  |
| other | Super Signal West Femto | Pierce | Cat. #: 34094 |  |
| other | RIPA lysis buffer | Cell Signaling Technology | Cat. #: 9806 |  |
| other | Sypro-Ruby | BioRad | Cat. #: 9806 |  |
